# Episodic memory network connectivity in temporal lobe epilepsy

**DOI:** 10.1111/epi.17370

**Published:** 2022-08-02

**Authors:** Marine Fleury, Sarah Buck, Lawrence P. Binding, Lorenzo Caciagli, Sjoerd B. Vos, Gavin P. Winston, Pamela J. Thompson, Matthias J. Koepp, John S. Duncan, Meneka K. Sidhu

**Affiliations:** ^1^ Department of Clinical and Experimental Epilepsy University College London Queen Square Institute of Neurology London UK; ^2^ MRI Unit Epilepsy Society Buckinghamshire UK; ^3^ Department of Computer Science, Centre for Medical Image Computing University College London London UK; ^4^ Department of Bioengineering University of Pennsylvania Philadelphia Pennsylvania USA; ^5^ Neuroradiological Academic Unit, University College London Queen Square Institute of Neurology University College London London UK; ^6^ Division of Neurology, Department of Medicine Queen's University Kingston Ontario Canada

**Keywords:** connectivity, epilepsy, memory, network, psychophysiological interaction

## Abstract

**Objective:**

Temporal lobe epilepsy (TLE) affects brain networks and is associated with impairment of episodic memory. Temporal and extratemporal reorganization of memory functions is described in functional magnetic resonance imaging (fMRI) studies. Functional reorganizations have been shown at the local activation level, but network‐level alterations have been underinvestigated. We aim to investigate the functional anatomy of memory networks using memory fMRI and determine how this relates to memory function in TLE.

**Methods:**

Ninety patients with unilateral TLE (43 left) and 29 controls performed a memory‐encoding fMRI paradigm of faces and words with subsequent out‐of‐scanner recognition test. Subsequent memory event‐related contrasts of words and faces remembered were generated. Psychophysiological interaction analysis investigated task‐associated changes in functional connectivity seeding from the mesial temporal lobes (MTLs). Correlations between changes in functional connectivity and clinical memory scores, epilepsy duration, age at epilepsy onset, and seizure frequency were investigated, and between connectivity supportive of better memory and disease burden. Connectivity differences between controls and TLE, and between TLE with and without hippocampal sclerosis, were explored using these confounds as regressors of no interest.

**Results:**

Compared to controls, TLE patients showed widespread decreased connectivity between bilateral MTLs and frontal lobes, and increased local connectivity between the anterior MTLs bilaterally. Increased intrinsic connectivity within the bilateral MTLs correlated with better out‐of‐scanner memory performance in both left and right TLE. Longer epilepsy duration and higher seizure frequency were associated with decreased connectivity between bilateral MTLs and left/right orbitofrontal cortex (OFC) and insula, connections supportive of memory functions. TLE due to hippocampal sclerosis was associated with greater connectivity disruption within the MTL and extratemporally.

**Significance:**

Connectivity analyses showed that TLE is associated with temporal and extratemporal memory network reorganization. Increased bilateral functional connectivity within the MTL and connectivity to OFC and insula are efficient, and are disrupted by greater disease burden.

**Key Points**

- Compared to controls, patients with TLE showed widespread memory network reorganization within the mesial temporal and frontal lobes
- Increased local functional connectivity between the MTLs bilaterally is supportive of memory functions in both left and right TLE
- Greater disease burden correlated with weaker connectivity within/to left MTL and within/from right MTL, connectivity supportive of verbal/visual memory
- Higher seizure frequency and longer epilepsy duration were associated with weaker connectivity in the OFC and insula, connectivity supportive of memory

## 1 INTRODUCTION

Temporal lobe epilepsy (TLE) is associated with widespread temporal and extratemporal neuronal dysfunction^1^ with corresponding impairments in episodic memory.^2^ Memory has been shown to be related to several clinical factors, including duration of epilepsy, age at onset of epilepsy, and seizure frequency,^3^, ^4^, ^5^, ^6^ resulting in a large variability in the degree of memory impairment among those with TLE.

In the past decade, compensatory memory reorganization of memory activations in TLE have been described^5^, ^7^, ^8^, ^9^, ^10^, ^11^; however, less is known about changes in functional connectivity within memory networks.

Using traditional activation‐based fMRI analyses, we previously showed a material‐specific pattern of reorganization in TLE.^11^ Nonetheless, in some people who had temporal lobe surgery, there was significant decline in verbal and visual episodic memory function that was not material‐specific.^12^ We hypothesize that although activation maxima may show hemispheric lateralization, the underlying memory network may be more widely distributed.

TLE is increasingly conceptualized as a network disorder.^13^, ^14^ Similarly, episodic memory is supported by a distributed network involving the hippocampus (HC) and neocortex.^15^, ^16^ Network analysis may provide a functional imaging biomarker for memory impairments that extend beyond the seizure focus.

Functional connectivity analysis of functional magnetic resonance imaging (fMRI) data identifies temporal correlations and connections between brain regions within a functional network.^17^ In those with TLE, there is disruption in functional connectivity within both temporal and extratemporal regions.^10^, ^18^, ^19^, ^20^, ^21^ Functional connectivity in relation to memory performance has only been explored using resting‐state fMRI, rather than task‐based memory analyses. Roger et al.^19^ used resting fMRI to show that increased connectivity within the left mesial temporal lobe (MTL) was associated with worse memory in people with left TLE (LTLE). Conversely, Barnett et al. showed that reduced resting functional connectivity between the left anterior HC and the default mode network was related to poor verbal memory in people with LTLE. Reduced connectivity between right posterior HC and posterior cingulate cortex was related to poor visual memory in people with right TLE (RTLE).^22^ These discordant findings are possibly due to functional connectivity being examined at rest, which reflects the subjects' state, rather than network changes specifically occurring during cognition.^23^

Functional connectivity studies of language networks have given good inference regarding the widespread network associated with language functions in epilepsy.^24^, ^25^ However, investigations of functional connectivity of memory networks in TLE are scarce.^26^, ^27^ These studies were performed using non‐material‐specific tasks, and the efficiency of reorganized networks were not explored.

The aim of this study was to investigate functional connectivity of memory in TLE by studying task‐specific changes in the memory encoding network that underlie subsequent successful memory. We used a verbal and visual memory fMRI task in TLE, implementing a seed‐based whole‐brain functional connectivity approach. Our specific aims were to examine (1) disruption of functional connectivity in TLE compared to controls, (2) clinical factors that affect reorganization of the memory network, and (3) whether these changes are related to memory test performance.

## 2 MATERIALS AND METHODS

We previously reported activation‐based fMRI analyses of verbal and visual memory activations in this cohort of participants using blocked and event‐related analyses.^11^ This study examines task‐based connectivity in these participants.

### 2.1 Subjects

We studied 90 people with medically refractory TLE (43 left) and 29 healthy controls, who were part of a previous cohort.^11^, ^12^ Patients underwent presurgical assessment at the National Hospital for Neurology and Neurosurgery between 2008 and 2013. All patients were on antiseizure medications. Controls were age‐ and sex‐matched to patients, and had no history of neurological or psychiatric disorder. Exclusion criteria included nonfluency in English, contraindication to MRI, and intelligence quotient (IQ) < 70. All three groups were comparable for demographic variables.

Handedness was measured using the Edinburgh Handedness Inventory.^28^ Seizure frequency was calculated as the average total number of focal impaired awareness seizures per month and focal to bilateral tonic–clonic seizures, collected from prospective seizure diaries at the time of scanning.

Prolonged video‐electroencephalographic telemetry confirmed ipsilateral seizure onset in all patients. All patients underwent structural MRI at 3.0 T. MRI identified hippocampal sclerosis (HS) in 57 patients (30 left), dysembryoplastic neuroepithelial tumor in 12 (six left), cavernoma in six (three left), and focal dysplasia in two (two left); seven patients (two left) were MRI negative with subsequent pathology showing gliosis or end folium gliosis, and six RTLE patients showed other abnormalities.

The study was approved by the National Hospital for Neurology and Neurosurgery and the University College London Institute of Neurology Joint Research Ethics Committee. Written informed consent according to the Declaration of Helsinki was obtained from each participant.

### 2.2 Neuropsychological tests

Participants underwent memory testing using verbal learning and visual learning subtests of the BIRT Memory and Information Processing Battery (BMIPB).^29^ For verbal learning, participants were asked to learn a list of 15 words, presented over five trials (maximum score = 75). For visual learning, participants were asked to learn a nine‐element design that was presented over five trials (maximum score = 45).

### 2.3 Magnetic resonance data acquisition

MRI studies were performed using a 3‐T General Electric Excite HDx MRI scanner. An 8‐channel radiofrequency (RF) receiver head array coil and body RF coil for transmission were used. For fMRI, gradient‐echo echo planar images were acquired, providing blood oxygen level‐dependent contrast. Each volume comprised 36 contiguous oblique axial slices, slice thickness = 2.5 mm (.3‐mm gap), field of view = 24 × 24 cm, echo time = 25 ms, repetition time = 2.75 s, matrix = 96 × 96 interpolated to 128 × 128 during image reconstruction, in‐plane resolution = 2.5, SENSitivity Encoding factor = 2.5.^11^ A total of 274 volumes were generated. The field of view was positioned to maximize coverage of the temporal and frontal lobes, with the slices aligned with the long axis of the HC on the sagittal view.

### 2.4 Memory paradigm

The memory encoding fMRI paradigm consisted of words and faces.^11^, ^12^ During scanning, participants were explicitly asked to try to memorize a total of 100 black‐and‐white photographs of nonfamous faces and 100 single concrete nouns. Each item was presented for 3 s, in 60‐s blocks. We used a different interstimulus interval (3 s) from our repetition time of 2.75 s to introduce jitter and facilitate random sampling. Each block consisted of 10 faces and 10 words followed by 15 s of crosshair fixation. A deep encoding task of pleasant/unpleasant decision was used for each item using a button box.

Forty minutes after scanning, participants performed an out‐of‐scanner recognition test, separately for words and faces. The same 100 items were intermixed with an additional 50 novel words/faces as foils and presented in random order and at the same speed as displayed inside the scanner. Participants indicated whether items were remembered, familiar, or novel using a button box. According to participants' responses, recognition accuracy was calculated based on items that were subsequently remembered versus rated as familiar or forgotten, for words and faces separately. Recognition accuracy consisted of a percentage of true positives subtracted by false positives.

### 2.5 Data analysis

Image preprocessing and connectivity using psychophysiological interaction (PPI) were performed using Statistical Parametric Mapping (SPM) version 12 (http://www.fil.ion.ucl.ac.uk/spm/). The imaging time series of each subject was realigned using the mean image as a reference to correct for motion artifact, normalized into standard anatomical Montreal Neurological Institute (MNI) space (using a scanner‐specific template created from 30 healthy control subjects, 15 patients with left HS and 15 patients with right HS, using the high‐resolution whole brain echo planar image)^11^ and smoothed with a Gaussian kernel of 8 mm full width at half maximum.

A two‐level event‐related random‐effects analysis was performed.^11^ At the first level, event‐related analyses were done based on a blocked design experiment.^9^, ^11^ Activation patterns at encoding of stimuli that were subsequently remembered (i.e., successful trials) were investigated. For each subject, the delta function of successful trials was convolved with the canonical hemodynamic response function and its temporal derivative.^11^ Two regressors of interest were created; words remembered and faces remembered. Each subject's movement parameters were added as confounds. Contrast images were generated for each subject for verbal subsequent memory and visual subsequent memory, and were used for the connectivity analysis.

#### 2.5.1 Seed within the MTL

We previously showed significant memory encoding activations, for subsequent successful memory, within the HC, and also showed reorganization to involve the parahippocampal gyrus (PHG) and amygdala in people with TLE.^9^, ^11^ To ensure we captured meaningful task‐based connectivity, at an individual level, each participant's first‐level event‐related analysis was visually inspected at *p* < .01 uncorrected to ensure that there were activations within this selected peak region.

MTL seed selection was based on a combined group maximum (i.e., in controls, LTLE and RTLE) within an MTL mask for the subsequent memory contrasts. The MTL mask incorporated the HC, PHG, and amygdala and determined the anatomical spatial extent for seed selection. Four seed regions were yielded: left and right MTL peak activation for words and faces remembered. Both left MTL seeds for words and faces remembered lay in the left anterior HC (*p* < .001, familywise error [FWE] corrected), and the right MTL seeds for faces and words remembered lay in the right anterior and middle HC (*p* < .001, FWE corrected), respectively (Table 1).

**TABLE 1:** MTL seeds chosen for psychophysiological interaction event‐related analyses on verbal and visual memory fMRI tasks

		T‐value	*z*‐value	Coordinates	*p*FWE	Region
Left MTL seeds	Faces remembered	11.81	>8	−18, −8, −18	*p* < .001	Left anterior HC
	Words remembered	10.75	>8	−26, −14, −16	*p* < .001	Left anterior HC
Right MTL seeds	Faces remembered	11.88	>8	18, −6, −16	*p* < .001	Right anterior HC
	Words remembered	7.44	6.70	32, −20, −8	*p* < .001	Right middle HC

*Note: p*FWE refers to activations reported at threshold *p* < .05, corrected for multiple comparisons using FWE rate.

Abbreviations: FWE, familywise error; HC, hippocampus; MTL, mesial temporal lobe.

#### 2.5.2 Functional connectivity analysis using psychophysiological interaction

We conducted a region of interest (ROI)‐to‐whole‐brain connectivity analysis.^24^, ^30^, ^31^, ^32^ PPI analysis was used to assess task‐related functional connectivity between brain regions, during encoding of subsequent successful memory.^33^ The seed region was identified as above. For each individual, the time series of a sphere of 6‐mm radius around this peak voxel was extracted from the images.

The PPI model included three regressors: (1) main effect of the seed region (i.e., the functional connectivity); (2) main effect of the task (encoding of subsequently remembered words or faces, separately); and (3) interaction between the two, representing a task‐modulated change in functional connectivity.^17^ This yielded a seed to voxelwise whole‐brain analysis for each participant.

Areas functionally coupled to the MTL seed region (left and right separately) were examined across groups with one‐sample *t*‐tests for each task, and also compared across groups with three‐way analyses of covariance (ANCOVAs). Group‐level PPI activations from the one‐sample *t*‐tests are reported at a threshold of *p* < .05, corrected for multiple comparisons (FWE) across the whole brain, unless otherwise stated. From the ANCOVAs, MTL functional connectivity is reported at *p* < .05 surviving correction for multiple comparisons (FWE) within a 6‐mm‐radius sphere, and neocortical connectivity is reported at an exploratory threshold of *p* < .001, uncorrected.^34^, ^35^

#### 2.5.3 Statistical analyses

##### Functional connectivity based on event‐related PPI

We conducted 3 one‐sample *t*‐tests to investigate the functional connectivity of the successful memory network, across both memory tasks for each MTL seed, separately in healthy controls, in LTLE, and in RTLE. Group comparison was done by running three‐way ANCOVAs. Memory scores and clinical variables (epilepsy duration, age at onset, and seizure frequency) were added as regressors of no interest (i.e., all regressors added in each three‐way ANCOVA) to assess differences in PPI between controls and both TLE groups, in relation to effect of TLE pathology on the memory network. These analyses were separately conducted for memory fMRI of faces and words subsequently remembered, and each was conducted with left and right MTL seeds separately.

Group comparison between patients with and without HS was carried out by running two‐sample *t*‐tests, in which memory scores and clinical factors were added as regressors of no interest, to investigate the effect of HS pathology on connectivity patterns in LTLE and RTLE.

##### Correlations of functional connectivity with out‐of‐scanner memory scores

We assessed correlations of areas of verbal and visual memory functional connectivity with out‐of‐scanner memory performance.^9^ Positive and negative correlations were examined using verbal and visual learning scores from the BMIPB, used as continuous variables in one‐sample *t*‐tests. These analyses examine the efficiency of functional connectivity to support memory function, and were conducted separately for encoding of faces and words subsequently remembered, and for left and right MTL seeds.

##### Correlation between functional reorganization in TLE and memory scores

We conducted three‐way ANCOVAs for each MTL seed and each task, to investigate which difference in functional connectivity between TLE and controls was supportive of memory functions. In each ANCOVA, individual memory scores were added as continuous regressor of interest. Group comparisons were performed separately for memory fMRI of faces and words subsequently remembered, and with left and right MTL seeds separately.

##### Correlation of functional connectivity with clinical factors

We assessed correlations between functional connectivity and clinical factors: epilepsy onset, epilepsy duration, and seizure frequency. Due to the intrinsic relationship between epilepsy duration and onset, these factors were controlled for by each other. For faces and words subsequently remembered, and for left and right MTL seeds, positive and negative correlations were examined in one‐sample *t*‐tests for each covariate of interest, used as continuous variable.^24^, ^36^

##### Correlation between connectivity supportive of memory performance and disease burden (seizure frequency and epilepsy duration)

We explored the relationship between functional connectivity that is supportive of memory functions and factors of disease burden: epilepsy duration and seizure frequency. To do so, we conducted positive and negative correlations in one‐sample *t*‐tests for each covariate of interest, whereby (1) either epilepsy duration or seizure frequency, and (2) memory scores were used as continuous variables. In each TLE group, the effect of disease burden on efficient connectivity was probed via correlations between longer/shorter epilepsy duration and better memory performance, and between higher/lower seizure frequency and better memory performance.

##### Statistical thresholds

Results from the one‐sample *t*‐tests of functional connectivity are reported corrected for multiple comparisons using FWE rate. For the rest of the above analyses, MTL connectivity is reported corrected for multiple comparison (FWE) using a small volume correction within a sphere radius of 6 mm (*p* < .05, FWE corrected), due to the greater specificity of the contrasts.^11^

Extratemporal connectivity is reported at an exploratory threshold of *p* < .001, uncorrected, in keeping with previous fMRI studies of memory, where at the cluster and network levels, group differences and correlations have been explored.^11^, ^12^, ^34^, ^35^

## 3 RESULTS

### 3.1 Subjects

Details on demographic and clinical variables are shown in Table 2, and out‐of‐scanner recognition accuracy is reported in Table S1. In each group, there was no significant difference between the proportion of male and female and between left‐ and right‐handed subjects, and in the proportion of TLE with HS and without HS in LTLE and RTLE (Pearson chi‐squared). Kruskal–Wallis test revealed no significant difference between groups in age and neither did Mann–Whitney *U*‐tests for age at onset and frequency of seizures. Analysis of variance (ANOVA) showed a significant between‐group difference in full scale IQ; therefore, post hoc *t*‐tests were performed, which showed that full scale IQ scores were significantly lower in LTLE than in controls (*p* < .001) and in RTLE than in controls (*p* = .001). There was no significant difference in memory scores between LTLE and RTLE. For memory scores, one‐way ANOVAs were performed to provide significance of difference between scores of the three groups at *p* < .05. Post hoc *t*‐tests showed that verbal and visual learning scores were significantly lower in LTLE than in controls (*p* < .001) and in RTLE than in controls (*p* < .001). There was no significant difference between scores of LTLE and RTLE for verbal and visual learning. Two‐sample *t‐*tests revealed that learning scores in RTLE with HS were significantly lower than in those without HS (*p* = .005). There was no significant difference in verbal learning scores in RTLE with HS compared to those without HS, nor for verbal and visual learning scores in LTLE with HS compared to those without HS.

**TABLE 2:** Demographic and clinical data and memory scores for controls and patients

	Age, years (IQR)	Sex, F/M	Handedness, L/R	FSIQ (IQR)	Age at onset, years (IQR)	FS monthly (IQR)	Verbal learning (SD)	Visual learning (SD)	Proportion of TLE with HS/total number
NC	37 (23)	12/17	5/24	111 (10)	NA	NA	55.5 (12)	40.5 (6)	NA/29
Left TLE	38 (18.25)	24/19	5/38	98 (12)	16 (16.5)	5 (8.75)	46 (19)	35 (9.5)	30/43
Right TLE	40 (16)	35/12	11/36	101 (13)	16.5 (13.75)	6 (9.5)	45 (10.75)	32 (13)	27/47
*p*	*p* = .936	*p* = .146	*p* = .342	*p* < .001	*p* = .687	*p* = .866	*p* < .001	*p* < .001	*p* = .226

*Note:* Age, FSIQ, and age at onset of epilepsy are shown as median and IQR. Verbal learning and visual learning are reported as mean and SD. FS with impaired awareness and focal to bilateral tonic–clonic seizures are shown as median and interquartile range. Probability values provide significance of difference between the three groups at *p* < .05.

Abbreviations: F, female; FS, focal seizures; FSIQ, full‐scale intelligence quotient; HS, hippocampal sclerosis; IQR, interquartile range; L, left; M, male; NA, not applicable; NC, normal controls; R, right; TLE, temporal lobe epilepsy.

### 3.2 Functional connectivity of memory

For each memory fMRI task, we conducted one‐sample *t‐*tests in each group to investigate the functional connectivity in controls, LTLE, and RTLE. Results (Table 3) are reported at *p* < .05, FWE correction, unless otherwise stated.

**TABLE 3:** Functional connectivity in controls and in patients with left and right TLE (one‐sample *t*‐tests)

		Controls	Left TLE	Right TLE
Visual memory	Left MTL	Left sup temporal pole −48, 22, −16 *p*FWE < .001 Left sup TG −58, 4, −14 *p*FWE = .007 Left mid TG −48, −16, −18 *p*FWE = .001 Left sup medial FG 0, 60, 0 *p*FWE < .001 Left inf FG, triangular part −40, 24, 20 *p*FWE = .002 Left inf PG −22, −52, 52 *p*FWE = .028 Left posterior HC −18, −32, −2 *p* = .041 Left posterior PHG −26, −38, −14 *p*FWE = .006 Left posterior FFG −24, −40, −14 *p*FWE = .003 Right posterior PHG 20, −44, −6 *p*FWE = .006 Right posterior FFG 28, −40, −14 *p*FWE = .019 Right sup temporal pole 56, 14, −8 *p*FWE = .001 Right mid TG 58, −18, −16 *p*FWE < .001 Right inf FG, orbital part 46, 32, −8 *p*FWE = .040 Right rolandic operculum 50, −12, 14 *p*FWE = .033 Right posterior OFC 46, 24, −16 *p*FWE = .029 Right supramarginal gyrus 54, −26, 28 *p*FWE < .001	Left sup TG −64, −18, 8 *p*FWE = .038 Threshold *p* < .05, FWE using a 6‐mm sphere correction Left anterior HC ‐22, −18, −14 *p* = .002^a^ Left posterior FFG −24, −38, −22 *p* = .010^a^ Right mid HC 22, −24, −8 *p* < .001^a^ Right posterior HC 24, −34, 2 *p* < .001^a^ Right posterior FFG 26, −34, −34 *p* = .007^a^ Right amygdala 20, 6, −18 *p* = .008^a^	Left sup TG −52, −24, 4 *p*FWE = .009 Left mid TG −46, −56, 18 *p*FWE = .009 Right sup TG 66, −26, 6 *p*FWE = .009 Right mid TG 64, −48, 0 *p*FWE = .001 Right supramarginal gyrus 66, −24, 26 *p*FWE = .005 Threshold *p* < .05, FWE using a 6‐mm sphere correction Left anterior PHG −28, −22, −22 *p* = .002^a^ Right anterior PHG 20, −6, −32 *p* = .001^a^ Right posterior FFG 26, −36, −12 *p* = .009^a^ Right posterior PHG 28, −28, −16 *p* = .006^a^
	Right MTL	Left inf FG, triangular part −40, 42, 12 *p*FWE = .004 Left sup TG −58, −28, 4 *p*FWE = .018 Left mid TG −48, −24, −2 *p*FWE = .039 Left posterior HC ‐12, −34, 8 *p*FWE = .009 Left posterior PHG −26, −38, −14 *p*FWE = .046 Left posterior FFG −26, −42, −10 *p*FWE = .029 Right posterior FFG 20, −44, −6 *p*FWE = .039 Right posterior HC 12, −38, 8 *p*FWE = .006 Right inf FG, triangular part 54, 28, 2 *p*FWE = .030 Right sup TG 54, −12, −2 *p*FWE < .001 Right mid TG 58, −26, −12 *p*FWE < .001	Left posterior FFG −24, −54, 6 *p*FWE < .001 Right posterior FFG 20, −46, −10 *p*FWE < .001 Right mid TG 54, −38, 2 *p*FWE = .001 Right supramarginal gyrus 58, −30, 32 *p*FWE = .021	Left mid TG −54, −70, 20 *p*FWE = .023 Right sup TG 64, −18, 10 *p*FWE = .016 Right mid TG 46, −54, 20 *p*FWE = .020 Right supramarginal gyrus 58, 48, 24 *p*FWE = .023 Threshold *p* < .05 FWE, using a 6‐mm sphere correction Left anterior HC ‐36, −18, −10 *p* < .003^a^ Right anterior HC 34, −14, −10 *p* = .001^a^ Right anterior PHG 24, −8, −34 *p* < .001^a^ Right posterior PHG 24, −22, −18 *p* = .006^a^
Verbal memory	Left MTL	Left sup temporal pole −48, 22, −16 *p*FWE = .002 Left mid TG −50, −20, −18 *p*FWE = .023 Left sup medial FG 0, 58, 2 *p*FWE = .023 Left inf FG, triangular part −42, 24, 22 *p*FWE = .033 Left midposterior PHG −26, −22, −20 *p*FWE = .001 Left midanterior HC ‐28, −20, −12 *p*FWE = .006 Left midanterior FFG −32, −28, −18 *p*FWE = .006 Right midanterior FFG 26, −32, −16 *p*FWE = .034 Right sup temporal pole 56, 14, −8 *p*FWE = .001 Right sup TG 50, −20, −4 *p*FWE = .035 Right mid TG 64, −18, −8 *p*FWE < .001	Threshold *p* < .05 FWE, with 6‐mm sphere correction Left midanterior PHG −24, −22, −18 *p* = .009^a^	Left mid TG −60, −14, −4 *p*FWE < .001 Right mid TG 66, −28, −8 *p*FWE = .003 Threshold *p* < .05 FWE, using a 6‐mm sphere correction Left posterior PHG −26, −28, −28 *p* = .002^a^ Left midposterior HC ‐26, −24, −16 *p* = .003^a^ Left posterior FFG −32, −34, −20 *p* = .007^a^
	Right MTL	Left mid TG −64, −52, 0 *p*FWE = .001 Left supramarginal gyrus −56, −54, 26 *p*FWE < .001 Left inf PG −36, −80, 40 *p*FWE < .001 Right sup temporal pole 54, 18, −8 *p*FWE = .003 Right sup TG 60, −50, 18 *p*FWE = .001 Right inf TG 64, −46, −10 *p*FWE = .027 Right supramarginal gyrus 58, −42, 26 *p*FWE = .001 Threshold *p* < .05 FWE using a 6‐mm sphere correction Left anterior HC −24, −20, −20 *p* < .001^a^ Left posterior FFG −26, −40, −16 *p* = .002^a^	Left sup TG −56, −24, 8 *p*FWE = .024 Threshold *p* < .05 FWE using a 6‐mm sphere correction Left posterior FFG −20, −50, −12 *p* = .015^a^ Right amygdala 20, 6, −18 *p* = .017^a^ Right middle HC 26, −24, −12 *p* = .017^a^	Right mid TG 56, −4, −24 *p*FWE = .006 Threshold *p* < .05 FWE, using a 6‐mm sphere correction Left posterior PHG −26, −26, −18 *p* = .004^a^ Right midposterior HC 28, −24, −14 *p* = .002^a^ Right posterior PHG 26, −22, −18 *p* = .002^a^

Abbreviations: FFG, fusiform gyrus; FG, frontal gyrus; FWE, corrected for multiple comparisons using familywise error rate; HC, hippocampus; inf, inferior; MTL, mesial temporal lobe; OFC, orbitofrontal cortex; PG, parietal gyrus; PHG, parahippocampal gyrus; sup, superior; TG, temporal gyrus; TLE, temporal lobe epilepsy.

^a^ FWE correction within a small volume correction of 6 mm.

#### 3.2.1 Controls

**FIGURE 1:**
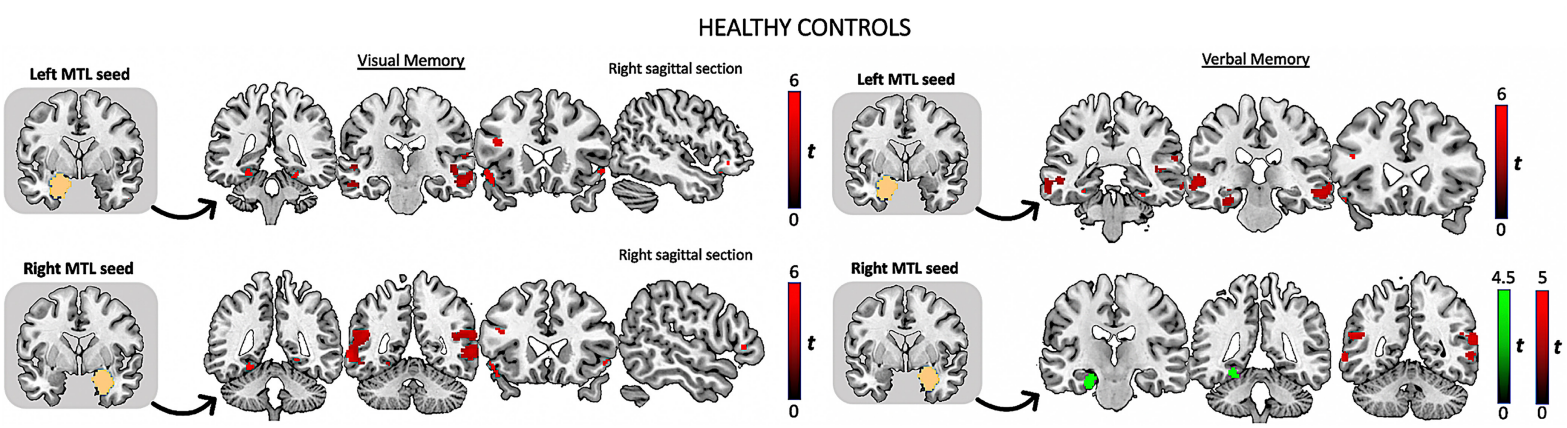
Task‐modulated changes in functional connectivity in healthy controls, seeding from (orange) the left mesial temporal lobe (MTL) and right MTL, for visual and verbal memory functional magnetic resonance imaging tasks. All images (coronal and sagittal sections) represent T‐value maps generated in Statistical Parametric Mapping that are overlaid onto a structural image (in Montreal Neurological Institute space) for visual localization purposes. Only significant psychophysiological interaction activations at the specified threshold are included. For the best representation of an efficient network on an individual basis, the peak voxel for seed selection was taken within the MTL region. Red clusters represent functional connectivity at threshold *p* < .05 corrected for multiple comparisons (familywise error [FWE]) at the global whole brain level, whereas green clusters represent functional connectivity between the right MTL seed and the left MTL shown at *p* < .05 corrected for multiple comparisons (FWE) using small volume correction within a 6‐mm‐radius sphere

On the visual memory task (Figure 1), healthy controls showed significant connectivity between the peak activation within each MTL seed and bilateral inferior frontal gyrus (IFG), bilateral middle/superior temporal gyrus (TG), bilateral posterior fusiform gyrus (FFG), and either bilateral posterior PHG or HC. There was significant connectivity between the left MTL seed and right orbitofrontal cortex (OFC). For verbal memory, seeding from either the left or right MTL seeds, there was significant connectivity with bilateral middle TG. Seeding from the left MTL, healthy controls displayed significant connectivity with the left IFG, and left anterior/posterior HC, PHG, and bilateral anterior FFG. At lower threshold (*p* < .05 surviving correction for multiple comparison [FWE] within a 6‐mm sphere), verbal memory was associated with functional connectivity between the right MTL seed and left anterior/posterior HC and FFG.

#### 3.2.2 Left TLE

On the visual memory task in LTLE, there was significant connectivity between the left MTL seed and left superior TG, and between the right MTL seed and right middle TG and supramarginal gyrus. At lower threshold than reported in controls (*p* < .05 surviving correction for multiple comparison [FWE] within a 6‐mm sphere), there was significant connectivity between the peak activation within each MTL seed and bilateral anterior/posterior HC and/or FFG. On the verbal memory task, seeding from the right MTL, there was significant connectivity with the left superior TG. At threshold *p* < .05 surviving FWE using a 6‐mm sphere correction, LTLE showed connectivity between each MTL seed and left anterior/posterior PHG/FFG, and between the right MTL seed and right HC and amygdala.

#### 3.2.3 Right TLE

On the visual memory task seeding from each MTL seed, patients with RTLE showed significant connectivity with bilateral middle/superior TG and right supramarginal gyrus, and at threshold *p* < .05 surviving a 6‐mm sphere correction (FWE), there was connectivity with bilateral anterior HC/PHG. For verbal memory, there was significant connectivity between the left/right MTL seed's peak voxel and the bilateral/right middle TG. At *p* < .05 surviving FWE using a 6‐mm sphere correction, RTLE displayed connectivity between the left MTL seed and left posterior PHG, HC, and FFG, and between the right MTL seed and bilateral posterior PHG and right posterior HC.

### 3.3 Functional connectivity of memory in TLE compared to controls

**FIGURE 2:**
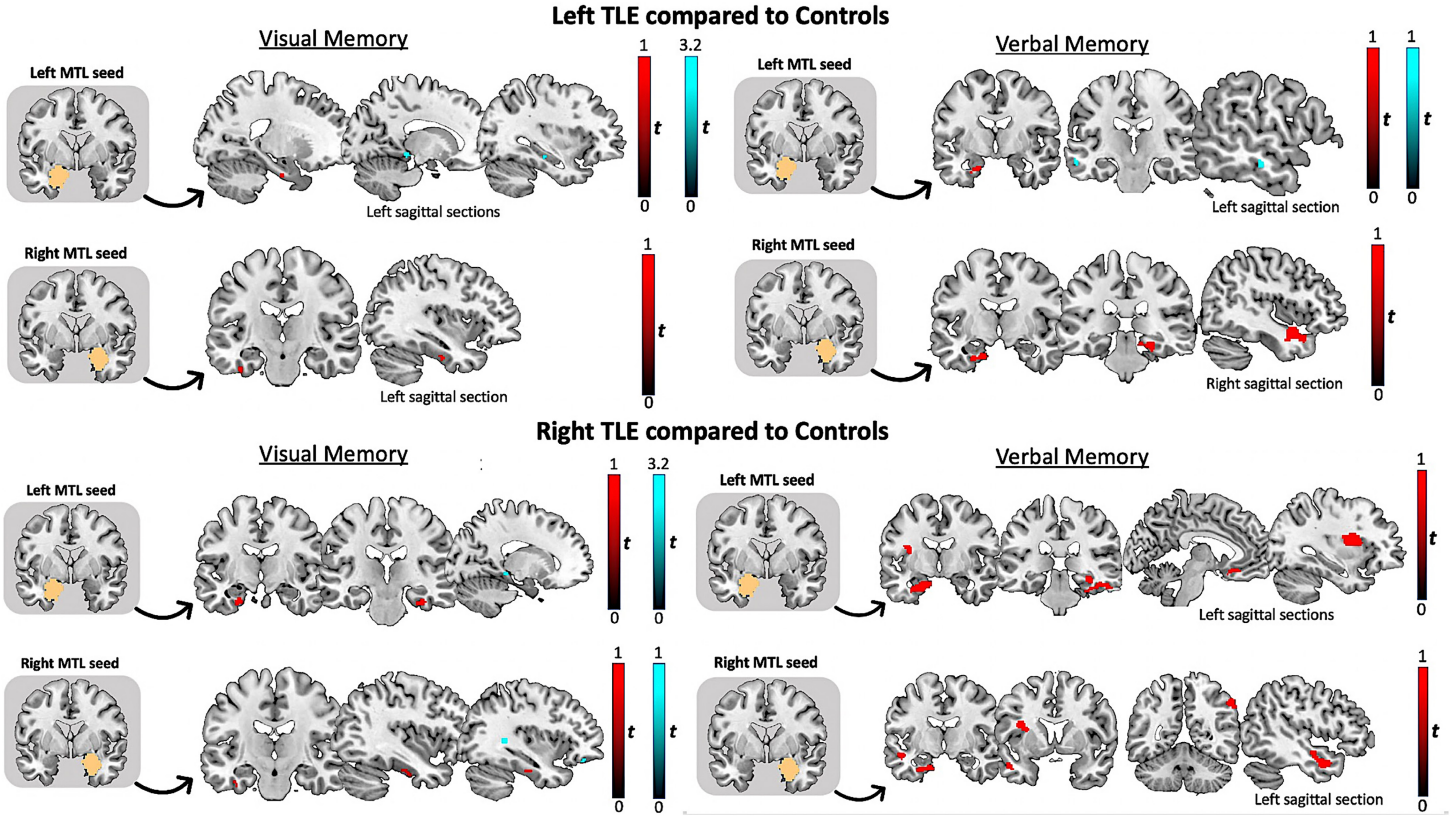
Task‐modulated changes in functional connectivity in left and right temporal lobe epilepsy (TLE) compared to controls, seeding from (orange) the left and right mesial temporal lobes (MTLs), for visual and verbal memory functional magnetic resonance imaging tasks. All images (coronal and sagittal sections) represent T‐value maps generated in Statistical Parametric Mapping that are overlayed onto a structural image (all in Montreal Neurological Institute space) for visual localization purposes. Significant MTL connectivity is visualized at *p* < .05, corrected for multiple comparisons (familywise error), using a 6‐mm‐radius sphere, and neocortical connectivity at *p* < .001, uncorrected. For the best representation of an efficient network on an individual basis, the peak voxel for seed selection was taken within the MTL region to reflect variations in activation maxima on single‐subject activation maps. Red clusters represent increased functional connectivity, and cyan clusters represent decreased connectivity in TLE compared to controls

We conducted three‐way ANCOVAs for each MTL seed and each task, to investigate differences in functional connectivity between each TLE group and controls (Table 4 and Figure 2). Age at onset of epilepsy, duration of epilepsy, seizure frequency, and memory performance are associated with memory network reorganization. These ANCOVAs were therefore performed with these parameters as regressors of no interest to study disease‐specific memory network reorganization.

**TABLE 4:** Functional connectivity in patients compared to controls (three‐way analyses of covariance, with memory scores and clinical variables as covariates)

		NC > LTLE	LTLE > NC	NC > RTLE	RTLE > NC
Visual memory	Left MTL	Left posterior PHG −16, −32 −6 *p* = .016^a^ Left midposterior HC −34, −24, −16 *p* = .032^a^	Left anterior PHG −24, −10, −30 *p* = .040^a^	Left posterior PHG −16, −32, −8 *p* = .014^a^	Left anterior PHG −24, −10, −30 *p* = .007^a^ Right anterior PHG 36, −22 −24 *p* = .021^a^
	Right MTL	None	Left anterior FFG −40, −16, −30 *p* = .029^a^	Left OFC, lateral part −38, 40, −16 *p* = .001^a^ Left posterior PHG −34, −44, 6 *p* = .009^a^	Left inf TG −38, −18, −28 *p* = .001 Left anterior FFG −36, −16, −30 *p* = .041^a^
Verbal memory	Left MTL	Left mid TG −58, −18, −16 *p* = .001^a^	Left anterior PHG −20, −8, −28 *p* = .022^a^	None	Left insula −36, 2, −18 *p* = .001 Left medial orbital FG −4, 24, −14 *p* = .001 Left anterior PHG −22, −10, −28 *p* = .013^a^ Left anterior HC ‐14, −4, −22 *p* = .043^a^ Right posterior PHG 32, −30, −12 *p* = .049^a^ Right inf TG 46, −38, −18 *p* = .001
	Right MTL	None	Left anterior PHG −16, −10, −28 *p* = .010^a^ Right posterior PHG 26, −28, −18 *p* = .007^a^ Right sup temporal pole 40, 16, −24 *p* = .001.	None	Left mid TG −44, 2, −28 *p* < .001 Left insula −34, 10, 16 *p* = .001 Left anterior PHG −26, −8, −32 *p* = .009^a^ Right inf PG 58, −54, 44 *p* < .001

Abbreviations: FFG, fusiform gyrus; FG, frontal gyrus; HC, hippocampus; inf, inferior; LTLE, left TLE; MTL, mesial temporal lobe; NC, normal controls; OFC, orbitofrontal cortex; PG, parietal gyrus; PHG, parahippocampal gyrus; RTLE, right TLE; sup, superior; TG, temporal gyrus; TLE, temporal lobe epilepsy.

^a^ Familywise error correction within a small volume correction of 6 mm for MTL functional connectivity. Exploratory neocortical functional connectivity is shown at *p* < .001, uncorrected.

Memory functional connectivity was compared between LTLE and RTLE with and without HS (Table 5). To examine the true effect of pathology, this was performed with the clinical variables described above as regressors of no interest. Differences in MTL functional connectivity are reported at *p* < .05 surviving correction for multiple comparison (FWE) within a 6‐mm‐radius sphere, and neocortical connectivity at an exploratory threshold of *p* < .001, uncorrected (see details in Section 2.5.3).

**TABLE 5:** Differences in functional connectivity between people with HS compared to without HS (analyses of covariance, with clinical variables and memory scores as covariates)

		Left TLE		Right TLE	
		HS > no HS	HS < no HS	HS > no HS	HS < no HS
Visual memory	Left MTL	Left inf PG −34, −52, 38 *p* = .001 Left mid TG −52, −8, −14 *p* = .001 Right mid TG 58, −66, 16 *p* = .001 Right anterior PHG 22, −12, −28 *p* = .004^a^	None	Left posterior PHG −12, −38, −6 *p* = .015^a^ Left posterior FFG −28, −30, −22 *p* = .030^a^	Left sup TG −52, 2, −2 *p* = .001 Right supramarginal 62, −22, 46 *p* = .001 Right sup TG 58, −8, −10 *p* < .001
	Right MTL	Left mid TG −56, −6, −20 *p* < .001 Left anterior HC −36, −14, −12 *p* = .003^a^ Right anterior HC 38, −8, −20 *p* = .006^a^ Right anterior PHG 24, −6, −28 *p* = .024^a^ Right amygdala 32, 0, −28 *p* = .025^a^ Right anterior FFG *p* = .037^a^	None	Right posterior FFG 36, −46, −10 *p* = .049^a^	Left insula −28, 18, −20 *p* < .001 Right sup TG 58, −10, −10 *p* < .001
Verbal memory	Left MTL	None	Right posterior HC 38, −26, −12 *p* = .029^a^	None	None
	Right MTL	Left anterior PHG −8, −6, −28 *p* = .012^a^ Left mid FG −54, 20, 36 *p* < .001 Left mid TG −58, −4, −20 *p* < .001 Left Inf TG −42, −2, −34 *p* = .001 Right sup FG 34, 58, 14 *p* = .001	Left supramarginal −66, −36, 34 *p* = .001 Right Rolandic operculum 42, −24, 18 *p* < .001 Right sup TG 60, −34, 12 *p* < .001	Right inf PG 52, −54, 40 *p* = .001	Right sup TG 58, −14, −8 *p* = .001

Abbreviations: FFG, fusiform gyrus; FG, frontal gyrus; HC, hippocampus; HS, hippocampal sclerosis; inf, inferior; MTL, mesial temporal lobe; PG, parietal gyrus; PHG, parahippocampal gyrus; sup, superior; TG, temporal gyrus; TLE, temporal lobe epilepsy.

^a^ Familywise error correction within a small volume correction of 6 mm for MTL functional connectivity. Exploratory neocortical functional connectivity is shown at *p* < .001, uncorrected.

#### 3.3.1 Left TLE

On both verbal and visual memory tasks, LTLE showed increased intrinsic MTL connectivity (i.e., connectivity observed between MTL regions) between the peak voxel within both MTL seeds and left anterior PHG/FFG than did controls. For verbal memory, there was increased connectivity between the right MTL and bilateral anterior/posterior PHG in LTLE than in controls. For visual memory, patients with LTLE displayed decreased connectivity between the left MTL seed and left posterior HC and PHG than in controls.

##### Connectivity in LTLE due to HS compared to non‐HS‐related pathology

For visual and verbal memory, LTLE patients with HS, compared to those without HS, showed increased anterior MTL connectivity, as well as temporal and extratemporal connectivity. For visual memory in LTLE with HS compared to without HS, there was increased connectivity between the left MTL seed and right anterior PHG and bilateral middle TG, and between the right MTL seed and bilateral anterior HC, right anterior PHG/FFG and amygdala, and left middle TG.

For verbal memory, patients with HS compared to without HS displayed increased connectivity between the right MTL and left anterior PHG, left inferior and middle TG, and left and right middle/superior frontal gyrus (FG). Verbal memory was associated with reduced posterior MTL connectivity between the left MTL and right posterior HC in patients with HS compared to those without HS.

#### 3.3.2 Right TLE

For visual memory seeding from both MTL seeds, RTLE showed increased MTL connectivity with bilateral/left anterior PHG/FFG compared to controls. There was increased connectivity between the right MTL seed and left inferior TG. Compared to healthy controls, RTLE showed reduced functional connectivity between both MTL seeds and left posterior PHG, and between the right MTL seed and the left OFC. For verbal memory seeding from both MTL seeds, RTLE showed greater left frontotemporal connectivity than did controls. In RTLE, there was increased connectivity between both MTL seeds and left insula, between the right MTL seed and the left middle TG, and between the left MTL seed and left medial orbital FG, than in controls.

##### Connectivity in RTLE due to HS compared to non‐HS‐related pathology

For visual memory in RTLE due to HS compared to non‐HS, there was increased intrinsic posterior MTL connectivity between the left MTL seed and left posterior PHG/FFG, and between the right MTL seed and right posterior FFG. RTLE with HS also showed reduced bilateral temporal (i.e., bilateral/right superior TG and left insula) seeding from the left/right MTL than did patients without HS.

For verbal memory, there was increased connectivity between the right MTL seed and right inferior parietal gyrus (PG) and reduced connectivity between the right MTL seed and right superior TG, in RTLE due to HS compared to those without HS.

### 3.4 Relation between functional connectivity and memory performance

Both TLE groups had worse memory compared to controls, and there was a wide range of verbal and design learning scores. Some people with epilepsy had comparable scores to healthy controls. Using these scores as a continuous regressor, we performed a correlation between functional connectivity of verbal and visual memory fMRI activations and standard memory scores of verbal and design learning (Table 6). All MTL connectivity and extratemporal connectivity is reported as above (see Section 3.3).

**TABLE 6:** Correlations between functional connectivity and memory scores (one‐sample *t*‐tests with memory scores as continuous variable)

		Controls		Left TLE		Right TLE	
		Positive correlation: better memory	Negative correlation: worse memory	Positive correlation: better memory	Negative correlation: worse memory	Positive correlation: better memory	Negative correlation: worse memory
Visual memory	Left MTL	Left posterior HC −22, −26, −10 *p* = .014^a^ Left anterior HC −18, −12, −14 *p* = .043^a^ Right anterior HC 22, −16, −12 *p* = .010^a^ Right inf FG, triangular part 56, 34, 16 *p* = .001	None	Left sup TG −50, −42, 18 *p* < .001 Left midtemporal pole −32, 14, −36 *p* < .001 Left anterior PHG −12, 0–28 *p* = .014^a^	None	Left anterior PHG −24, −10, −30 *p* = .048^a^ Right posterior PHG 34, −32, −12 *p* = .022^a^	None
	Right MTL	None	Left inf TG −44, −10, −30 *p* = .001 Right anterior PHG 22, 2, −32 *p* = .010^a^	Left supramarginal −54, −42, 26 *p* < .001 Right sup temporal pole 40, 18, −32 *p* < .001 Right midtemporal pole 44, 16, −42 *p* < .001 Right amygdala *p* = .020^a^	None	Left posterior HC −18, −28, −12 *p* = .008^a^ Right anterior PHG 18, 4, −24 *p* = .022^a^ Right mid TG 60, −42, 4 *p* < .001	None
Verbal memory	Left MTL	Right posterior FFG 48, −40, −16 *p* = .042^a^ Left mid TG −38, −58, 2 *p* = .001	None	Right posterior HC 14, −34, 10 *p* = .023^a^	Right sup FG 30, 48, 6 *p* = .001	Right inf FG, triangular 52, 42, 8 *p* < .001	None
	Right MTL	Left insula −36, −32, 22 *p* < .001	None	Left mid TG −66, −34, −6 *p* < .001 Left anterior FFG −32, −24, −28 *p* = .004^a^ Right posterior PHG 30, −24, −24 *p* = .007^a^	None	Right insula 44, 4, −12 *p* < .001 Left anterior PHG −30, −12, −28 *p* = .050^a^ Left posterior PHG −14, −42, −4 *p* = .049^a^	Left sup TG −58, −40, 16 *p* = .001 Right supramarginal 52, −44, 24 *p* = .001

Abbreviations: FFG, fusiform gyrus; FG, frontal gyrus; HC, hippocampus; inf, inferior; MTL, mesial temporal lobe; PHG, parahippocampal gyrus; sup, superior; TG, temporal gyrus; TLE, temporal lobe epilepsy.

^a^ Familywise error correction within a small volume correction of 6 mm for MTL functional connectivity. Exploratory neocortical functional connectivity is shown at *p* < .001, uncorrected.

#### 3.4.1 Controls

For visual memory in controls, better performances were associated with connectivity between the left MTL and bilateral anterior/posterior HC. Worse memory was correlated with increased connectivity between the right MTL seed and left TG. For verbal memory in controls, higher scores were correlated with increased connectivity to the right posterior FFG and left middle TG, seeding from the left MTL, and seeding from the right MTL, with connectivity to the left insula.

#### 3.4.2 Left TLE

For verbal and visual memory, better memory performance was associated with increased intrinsic MTL connectivity; between the left MTL seed and left anterior PHG and right MTL seed and right amygdala for visual memory; and for verbal memory between the left MTL seed and right posterior HC and the right MTL seed and left anterior FFG/right posterior PHG. Seeding from either left or right MTL, increased temporal connectivity between the left middle/superior TG was associated with better visual/verbal memory.

For verbal memory in LTLE, increased connectivity between the left MTL seed and right superior FG was correlated with poorer performance. There was no negative correlation between visual memory performance and changes in connectivity seeding from either MTL.

#### 3.4.3 Right TLE

For both verbal and visual memory, seeding from both MTLs, increased connectivity to the anterior/posterior PHG was significantly correlated with better memory performance; between both MTLs and bilateral PHG for visual memory; and between the right MTL and left PHG for verbal memory.

### 3.5 Functional reorganization in TLE supportive of memory performance

We conducted three‐way ANCOVAs for each MTL seed and each task, to investigate which difference in functional connectivity between TLE and controls was supportive of memory functions (Table 7). In each ANCOVA, individual memory scores were added as continuous regressor of interest. All MTL connectivity and extratemporal connectivity are reported as above (see Section 3.3).

**TABLE 7:** Correlations between differences in functional connectivity and better memory performance (three‐way analyses of covariance, with individual memory scores as continuous regressor of interest)

		Negative correlation: LTLE < NC and high memory scores	Positive correlation: LTLE > NC and high memory scores	Negative correlation: RTLE < NC and high memory scores	Positive correlation: RTLE > NC and high memory scores
Visual memory	Left MTL	Left mid TG −48, −72, −22 *p* < .001 Right anterior PHG *p* = .014^a^ Right supramarginal G 48, −44, 26 *p* < .001 Right sup TG 46, −38, 12 *p* < .001	None	None	None
	Right MTL	Left mid TG −44, −72, 20 *p* < .001 Left supramarginal G −44, −48, 26 *p* < .001 Left Rolandic operculum −56, −8, 10 *p* < .001 Left anterior HC ‐36, −12, −20 *p* = .045^a^	Right amygdala 20, 4, −18 *p* = .036^a^	Left sup PG −12, −70, 44 *p* < .001	None
Verbal memory	Left MTL	Left posterior FFG −36, −28, −18 *p* = .009^a^ Left posterior PHG −28, −34, −10 *p* = .031^a^ Left mid TG −38, −62, 14 *p* = .001 Right mid TG 54, −54, 16 *p* < .001 Right supramarginal G 62, −40, 32 *p* = .001	None	Left supramarginal G −64, −24, 40 *p* = .001 Left posterior HC ‐28, −30, −6 *p* = .029^a^ Right posterior FFG 28, −42, −16 *p* = .003^a^ Right posterior PHG 24, −28, −20 *p* = .049^a^ Right mid TG 52, −26, −10 *p* = .001	None
	Right MTL	Left supramarginal G −44, −48, 26 *p* < .001 Left anterior HC −24, −16, −22 *p* = .024^a^ Right mid TG 64, −18, −8 *p* = .001	Left sup temporal pole −44, −2, −14 *p* < .001	Left posterior HC ‐22, −16, −18 *p* = .032^a^ Right sup TG 48, −36, 18 *p* = .001	None

Abbreviations: FFG, fusiform gyrus; G, gyrus; HC, hippocampus; LTLE, left TLE; MTL, mesial temporal lobe; NC, normal controls; PG, parietal gyrus; PHG, parahippocampal gyrus; RTLE, right TLE; sup, superior; TG, temporal gyrus; TLE, temporal lobe epilepsy.

^a^ Familywise error correction within a small volume correction of 6 mm for MTL functional connectivity. Exploratory neocortical functional connectivity is shown at *p* < .001, uncorrected.

#### 3.5.1 Left TLE

For visual memory, LTLE patients showed decreased connectivity supportive of memory functions compared to that in controls, from the left MTL seed to right anterior PHG, left/right middle/superior TG, and right supramarginal gyrus. LTLE patients displayed increased intrinsic MTL connectivity supportive of visual memory, between right MTL seed and right amygdala.

For verbal memory in LTLE compared to controls, there was decreased connectivity supportive of memory functions within/to the left MTL (between left MTL seed and left posterior FFG and PHG, and between right MTL seed and left anterior HC), and from the left MTL seed to bilateral middle TG and right supramarginal gyrus. LTLE patients compared to controls showed increased connectivity supportive of verbal memory from the right MTL seed to left superior temporal pole.

#### 3.5.2 Right TLE

For visual memory, RTLE patients showed decreased connectivity supportive of memory functions, from the right MTL seed to left superior PG, compared to controls. There was no increased connectivity that was significantly correlated with better visual memory performance in RTLE.

For verbal memory, RTLE patients displayed decreased connectivity supportive of memory functions between MTL seeds and left posterior HC and right middle/superior TG, and between left MTL seed and right posterior FFG and PHG. There was no increased connectivity compared to controls that was correlated to better verbal memory.

### 3.6 Clinical factors affecting functional connectivity

For verbal and visual memory tasks, and seeding from each MTL, correlations were performed between changes in functional connectivity and epilepsy duration controlling for epilepsy onset (Table 8), epilepsy onset controlling for epilepsy duration (Table 9), and seizure frequency (Table 10).

**TABLE 8:** Positive and negative correlations between preoperative functional connectivity and epilepsy duration, controlling for epilepsy onset (one‐sample *t*‐tests with epilepsy duration as continuous variable)

		Left TLE		Right TLE	
		Positive correlation: longer duration	Negative correlation: shorter duration	Positive correlation: longer duration	Negative correlation: shorter duration
Visual memory	Left MTL	None	None	None	Left medial orbital FG −2, 28, −14 *p* < .001 Left anterior PHG −24, −8, −30 *p* = .006^a^ Left anterior HC −16, −4, −22 *p* = .007^a^ Right anterior PHG 18, −12, −28 *p* = .011^a^ Right posterior PHG 30, −26, −24 *p* = .010^a^ Right mid FG 38, 48, −6 *p* < .001
	Right MTL	None	Left inf TG −42, −16, −22 *p* = .001	None	Left anterior HC −16, −4, −22 *p* = .019^a^ Left amygdala −20, −2, −20 *p* = .023^a^ Left posterior PHG −16, −24, −18 *p* = .005^a^ Right mid TG 58, −36, 0 *p* < .001 Right medial orbital FG 0, 24, −10 *p* < .001 Right anterior PHG 32, −16, −26 *p* = .043^a^ Right amygdala 22, 4, −14 *p* = .049^a^
Verbal memory	Left MTL	None	Left inf TG −42, −8, −28 *p* = .001 Left anterior PHG −12, 2, −18 *p* = .030^a^	None	Left anterior PHG −14, −4, −20 *p* = .009^a^ Right posterior PHG 38, −16, −26 *p* = .026^a^ Right posterior FFG 30, −28, −22 *p* = .044^a^ Right anterior OFC 22, 36, −8 *p* < .001
	Right MTL	None	None	None	Left inf FG, operculum −38, 10, 16 *p* = .001 Left anterior PHG −26, −10, −28 *p* = .001^a^ Right posterior PHG 26, −22, −20 *p* = .002^a^

Abbreviations: FFG, fusiform gyrus; FG, frontal gyrus; HC, hippocampus; inf, inferior; MTL, mesial temporal lobe; OFC, orbitofrontal cortex; PHG, parahippocampal gyrus; TG, temporal gyrus; TLE, temporal lobe epilepsy.

^a^ Familywise error correction within a small volume correction of 6 mm for MTL functional connectivity. Exploratory neocortical functional connectivity is shown at *p* < .001, uncorrected.

**TABLE 9:** Positive and negative correlations between functional connectivity and epilepsy onset, controlling for epilepsy duration (one‐sample *t*‐tests with epilepsy onset as continuous variable)

		Left TLE		Right TLE	
		Positive correlation: later onset	Negative correlation: earlier onset	Positive correlation: later onset	Negative correlation: earlier onset
Visual memory	Left MTL	Left mid FG −38, 56, −6 *p* < .001	Right posterior FFG 32, −34, −26 *p* = .050^a^ Right sup TG 62, −2, −10 *p* = .001	Left mid HC *p* = .048^a^	Right posterior PHG 28, −14, −26 *p* = .021^a^
	Right MTL	Left inf FG, operculum −58, 16, 14 *p* = .001 Left sup FG −20, 66, −2 *p* = .001	Left posterior FFG −36, −28, −22 *p* = .027^a^ Right mid TG 44, −20, −10 *p* < .001	None	Left inf FG, triangular part −36, 28, 20 *p* = .001 Right mid TG 48, −54, 8 *p* = .001 Right posterior PHG 28, −22, −24 *p* = .001^a^
Verbal memory	Left MTL	None	Left inf TG −44, −6, −28 *p* = .001 Right anterior FFG 42, −12, −34 *p* = .031^a^	Left mid TG −58, −26, −8 *p* < .001 Right sup TG 60, −18, 0 *p* = .001 Left posterior PHG −16, −34, −6 *p* = .040^a^	Right posterior PHG 26, −12, −26 *p* = .039^a^
	Right MTL	None	None	Left inf FG, triangular part −38, 32, 4 *p* < .001 Left inf FG, operculum −58, 14, 14 *p* = .001 Right mid FG 38, 48, −2 *p* < .001 Right mid TG 56, −20, −10 *p* = .001	Left anterior PHG −18, −10, −28 *p* = .003^a^ Right posterior PHG 24, −26, −28 *p* < .001^a^

Abbreviations: FFG, fusiform gyrus; FG, frontal gyrus; HC, hippocampus; inf, inferior; MTL, mesial temporal lobe; PHG, parahippocampal gyrus; sup, superior; TG, temporal gyrus; TLE, temporal lobe epilepsy.

^a^ Familywise error correction within a small volume correction of 6 mm for MTL functional connectivity. Exploratory neocortical functional connectivity is shown at *p* < .001, uncorrected.

**TABLE 10:** Positive and negative correlations between functional connectivity and seizure frequency (one‐sample *t*‐tests with seizure frequency as continuous variable)

		Left TLE		Right TLE	
		Positive correlation: higher sz freq	Negative correlation: lower sz freq	Positive correlation: higher sz freq	Negative correlation: lower sz freq
Visual memory	Left MTL	None	None	None	Left mid TG −48, −66, 14 *p* < .001 Left inf TG −52, −10, −26 *p* = .001
	Right MTL	None	None	None	Left inf TG −50, −8, −28 *p* < .001 Right sup TG 62, −44, 16 *p* < .001 Right mid FG 48, 46, 14 *p* < .001 Right amygdala 24, −4, −18 *p* = .038^a^ Right midanterior PHG 30, −18, −30 *p* = .041^a^
Verbal memory	Left MTL	Right posterior HC 34, −26, −4 *p* = .019^a^ Right anterior PHG 20, −8, −36 *p* = .024^a^	None	None	Left mid TG −48, −62, 12 *p* < .001 Right midanterior PHG 30, −18, −30 *p* = .019^a^
	Right MTL	None	None	Left posterior OFC −18, 8, −22 *p* < .001 Right insula 44, 0, −8 *p* < .001 Right anterior HC 36, −12, −26 *p* = .012^a^ Right posterior PHG 36, −34, −14 *p* = .035^a^	Left mid TG −52, −8, −26 *p* = .001 Left sup FG −22, 58, 4 *p* < .001 Left posterior HC ‐30, −32, 0 *p* = .019^a^ Right sup TG 62, −46, 16 *p* < .001 Right mid FG 48, 30, 22 *p* < .001 Right medial sup FG 8, 62, 0 *p* < .001

Abbreviations: FG, frontal gyrus; freq, frequency; HC, hippocampus; inf, inferior; MTL, mesial temporal lobe; OFC, orbitofrontal cortex; PHG, parahippocampal gyrus; sup, superior; sz, seizure; TG, temporal gyrus; TLE, temporal lobe epilepsy.

^a^ Familywise error correction within a small volume correction of 6 mm for MTL functional connectivity. Exploratory neocortical functional connectivity is shown at *p* < .001, uncorrected.

#### 3.6.1 Epilepsy duration

##### Left TLE

For visual and verbal memory, longer epilepsy duration significantly correlated with reduced connectivity between the right/left MTL seed and left inferior TG. For verbal memory, longer epilepsy duration was associated with weaker intrinsic MTL connectivity between the left MTL seed and left anterior PHG.

##### Right TLE

For both verbal and visual memory in RTLE, longer epilepsy duration was significantly associated with reduced bilateral MTL connectivity (primarily with the bilateral anterior/posterior PHG) seeding from both MTLs. Longer epilepsy duration was significantly correlated with weaker connectivity between the left MTL seed and right OFC and the right MTL seed and left IFG for verbal memory, and for visual memory between the left/right MTLs and left/right medial orbital FG.

#### 3.6.2 Age at epilepsy onset

##### Left TLE

For visual and verbal memory in LTLE, earlier age at onset was significantly correlated with stronger intrinsic MTL connectivity, between both MTLs and right/left posterior FFG for visual memory, and between left MTL and right anterior FFG for verbal memory. Earlier age at onset was associated with reduced ipsilesional extratemporal connectivity between left MTL and middle FG and between right MTL and inferior/superior FG.

##### Right TLE

For visual and verbal memory in RTLE, earlier age at onset was significantly associated with stronger connectivity between both MTL seeds and right posterior PHG. For verbal memory, earlier age at onset was correlated with reduced temporal connectivity between the left MTL seed and left posterior PHG, and left and right middle/superior TG, along with weaker extratemporal connectivity between the right MTL seed and left and right inferior/middle FG.

#### 3.6.3 Seizure frequency

##### Left TLE

For visual memory, there was no significant correlation between frequency of seizures and changes in functional connectivity seeding from both MTLs. For verbal memory, higher seizure burden was correlated with increased connectivity between the left MTL seed and right MTL (i.e., right posterior HC/anterior PHG).

##### Right TLE

For both verbal and visual memory, higher seizure frequency was correlated with weaker temporal (e.g., within the left inferior/middle TG, right superior TG) and extratemporal (e.g., right middle FG) connectivity, seeding from the left or right MTL. For verbal memory, RTLE with higher seizure burden showed weaker left OFC connectivity, seeding from the right MTL.

For visual memory in RTLE, higher seizure frequency was significantly associated with weaker connectivity between right/left MTL seeds and right anterior MTL (amygdala and/or anterior PHG). For verbal memory, greater seizure burden was correlated with reduced left posterior HC connectivity and increased connectivity to the right anterior HC/posterior PHG, seeding from the right MTL.

### 3.7 Relation between connectivity supportive of memory performance and disease burden (seizure frequency and epilepsy duration)

The effect of disease burden on efficient connectivity (i.e., supportive of memory functions) was probed via correlations between longer/shorter epilepsy duration and better memory performance, and between higher/lower seizure frequency and better memory performance (see Tables 11 and 12). All MTL connectivity and extratemporal connectivity are reported as above (see Section 3.3).

**TABLE 11:** Correlations between seizure frequency and better memory performance (one‐sample *t*‐tests with seizure frequency and memory scores as continuous variables)

		Left TLE		Right TLE	
		Higher frequency and performance	Lower frequency and higher performance	Higher frequency and performance	Lower frequency and higher performance
Visual memory	Left MTL	Left midposterior HC −32, −22, −12 *p* = .016^a^ Left mid PHG −24, −20, −24 *p* = .021^a^ Left anterior FFG −36, −20, −28 *p* = .047^a^ Left sup TG −50, −42, 18 *p* < .001 Left midtemporal pole −32, 14, −40 *p* < .001	Left midposterior HC ‐32, −22, −12 *p* < .001^a^ Left anterior PHG −12, 0, −28 *p* = .016^a^ Left anterior FFG −36, −18, −28 *p* = .026^a^ Left mid TG −62, −32, −4 *p* < .001	Right posterior PHG 34, −32, −12 *p* = .041^a^	Right posterior PHG 34, −32, −12 *p* = .033^a^ Left anterior PHG −24, −10, −28 *p* = .039^a^ Left anterior HC ‐30, −16, −18 *p* = .046^a^
	Right MTL	Right sup temporal pole 40, 18, −32, *p* < .001 Right sup TG 68, −32, 20 *p* < .001 Left posterior HC ‐20, −36, 4 *p* = .003^a^	Left inf PG −52, −42, 48 *p* = .001 Left supramarginal −54, −44, 26 *p* = .001 Right sup temporal pole 40, 18, −32 *p* < .001 Right midtemporal pole 46, 18, −40 *p* < .001 Right amygdala 22, 6, −18 *p* = .015^a^	Left posterior HC −18, −28, −12 *p* = .019^a^ Right anterior PHG 18, 2, −24 *p* = .029^a^ Right posterior PHG 34, −32, −12 *p* = .048^a^ Right mid TG 60, −42, 4 *p* = .001	Left posterior HC −20, −28, −12 *p* = .010^a^ Left sup PG −32, −48, 56 *p* = .001 Left inf PG −34, −34, 38 *p* < .001 Right Inf PG 42, −36, 48 *p* < .001 Right anterior PHG 16, 6, −24 *p* = .023^a^ Right sup TG 60, −48, 16 *p* < .001 Right mid TG 62, −40, 4 *p* < .001 Right supramarginal gyrus 58, −32, 50 *p* < .001 Right mid FG 34, 28, 18 *p* < .001
Verbal memory	Left MTL	Right sup TG 56, −22, 8 *p* < .001	Left supramarginal gyrus −42, −34, 26 *p* < .001 Left subcentral gyrus −40, −26, 20 *p* = .001	None	Right inf FG, triangular part, 52, 42, 8 *p* < .001 Left anterior FFG −34, −20, −30 *p* = .032^a^
	Right MTL	Left mid TG −66, −34, −4 *p* < .001 Right anterior PHG 30, −22, −22 *p* = −.24^a^	Left mid TG −56, −48, −2 *p* < .001 Left inf TG −38, −14, −32 *p* < .001 Left midanterior FFG −28, −26, −28 *p* = .013^a^ Right anterior FFG 36, −34, −36 *p* = .005^a^	Left medial OFC ‐2, 30, −12 *p* < .001 Left anterior PHG −30, −12, −28 *p* = .018^a^ Right anterior HC 36, −12, −24 *p* = .029^a^ Right posterior PHG 28, −26, −14 *p* = .034^a^ Right insula 42, 2, −8 *p* < .001 Right posterior OFC *p* < .001 Right sup TG 50, 20, −16 *p* = .001	None

Abbreviations: FFG, fusiform gyrus; FG, frontal gyrus; HC, hippocampus; inf, inferior; MTL, mesial temporal lobe; OFC, orbitofrontal cortex; PG, parietal gyrus; PHG, parahippocampal gyrus; sup, superior; TG, temporal gyrus; TLE, temporal lobe epilepsy.

^a^ Familywise error correction within a small volume correction of 6 mm for MTL functional connectivity. Exploratory neocortical functional connectivity is shown at *p* < .001, uncorrected.

**TABLE 12:** Correlations between epilepsy duration and better memory performance (one‐sample *t*‐tests with epilepsy duration and memory scores as continuous variables)

		Left TLE		Right TLE	
		Higher duration and performance	Lower duration and higher performance	Higher duration and performance	Lower duration and higher performance
Visual memory	Left MTL	Left midposterior HC −32, −22, −12 *p* = .001^a^ Left mid PHG −32, −24, −20 *p* = .020^a^ Left mid TG −42, −40, 6, *p* = .001	Left midposterior HC −32, −22, −12 *p* = .005^a^ Left anterior PHG −24, −22, −24 *p* = .029^a^ Left posterior FFG −28, −48, −6 *p* = .013^a^ Left sup TG −52, −44, 18 *p* < .001 Left mid TG −44, −52, 20 *p* < .001 Left midtemporal pole −32, 14, −38 *p* < .001 Right sup TG 60, −56, 20 *p* = .001 Right mid TG 44, −48, 18 *p* < .001	None	Left midanterior PHG −24, −10, −30 *p* = .018^a^ Right posterior PHG 34, −32, −12 *p* = .018^a^
	Right MTL	Left supramarginal gyrus −54, −22, 34 *p* < .001 Left inf PG −50, −38, 42 *p* = .001 Left mid TG −54, −62, 2 *p* = .001 Right anterior PHG 18, 2, −24 *p* = .021^a^ Right anterior FFG 28, 0, −42 *p* = .033^a^ Right midtemporal pole 42, 18, −40 *p* < .001	Left posterior HC −18, −38, 4 *p* = .007^a^ Left anterior PHG −18, −2, −26 *p* = .015^a^ Left insula −36, −20, −2 *p* = .001 Left mid FG −48, 12, 48 *p* = .001 Left supramarginal gyrus −54, −42, 26 *p* < .001 Left sup PG −38, −54, 62 *p* = .001 Right amygdala 22, 6, −16 *p* = .046^a^ Right sup temporal pole 40, 18, −32 *p* < .001 Right midtemporal pole 44, 16, −42 *p* = .001 Right sup TG 68, −34, 20 *p* < .001	Left midposterior HC 18, −30, −10 *p* = .017^a^	Left midposterior HC ‐18, −28, −12 *p* = .013^a^ Left anterior PHG −18, −8, −28 *p* = .040^a^ Right anterior PHG 18, 4, −24 *p* = .032^a^ Right mid TG 60, −42, 4 *p* < .001 Right mid FG 32, 28, 20 *p* = .001
Verbal memory	Left MTL	None	Left posterior HC ‐20, −40, 2 *p* = .009^a^ Right posterior FFG 34, −46, −4 *p* = .014^a^ Right posterior HC 28, −34, 4 *p* = .031^a^	Right anterior PHG 24, 10, −24 *p* = .027^a^	Left OFC medial part −8, 30, −12 *p* < .001 Left anterior PHG −12, −4, −20 *p* = .019^a^ Right midposterior HC 26, −24, −6 *p* = .043^a^ Right mid FG 44, 42, 8 *p* < .001
	Right MTL	Right anterior PHG 28, −16, −28 *p* = .027^a^ Right anterior FFG 26, −16, −32 *p* = .040^a^	Left midposterior FFG −26, −28, −26 *p* = .011^a^ Left anterior HC ‐20, −14, −14 *p* = .030^a^ Right mid PHG 32, −24, −24 *p* = .010^a^ Right midposterior HC 38, −20, −8 *p* = .001^a^ Right mid FFG 32, −26, −24 *p* = .008^a^	None	Left anterior PHG −28, −10, −28 *p* = .002^a^ Right posterior PHG 26, −24, −18 *p* = .015^a^ Right sup temporal pole 40, 16, −22 *p* < .001 Right insula 42, 4, −12 *p* < .001 Right OFC medial part 20, 12, −22 *p* = .001

Abbreviations: FFG, fusiform gyrus; FG, frontal gyrus; HC, hippocampus; inf, inferior; MTL, mesial temporal lobe; OFC, orbitofrontal cortex; PG, parietal gyrus; PHG, parahippocampal gyrus; sup, superior; TG, temporal gyrus; TLE, temporal lobe epilepsy.

^a^ Familywise error correction within a small volume correction of 6 mm for MTL functional connectivity. Exploratory neocortical functional connectivity is shown at *p* < .001, uncorrected.

#### 3.7.1 Left TLE

For visual memory in LTLE, regardless of the degree of disease burden (epilepsy duration and seizure frequency), connectivity between the left MTL seed and left posterior/anterior HC, PHG, and/or FFG and left temporal cortex (middle TG and/or temporal pole) was significantly correlated with better memory scores. LTLE with longer epilepsy duration showed decreased connectivity supportive of memory functions, between both MTL seeds and right temporal cortex (middle or superior TG/temporal pole), and between right MTL seed and left MTL (posterior/anterior HC/PHG) and frontal cortex (left insula and middle FG). LTLE with higher seizure frequency displayed decreased connectivity from right MTL seed to right amygdala and to the left PGs (left inferior parietal and supramarginal gyri).

For verbal memory in LTLE, intrinsic connectivity between right MTL seed and right anterior PHG and/or FFG was efficient regardless of seizure frequency or epilepsy duration. However, there was decreased connectivity supportive of verbal memory, from right MTL to left anterior FFG in LTLE with higher disease burden, and between both MTLs and bilateral posterior/anterior HC in LTLE with longer duration. LTLE with greater seizure burden showed significantly weaker connectivity supportive of verbal memory, from the left MTL seed to left supramarginal and subcentral gyri.

#### 3.7.2 Right TLE

For visual memory, regardless of the degree of disease burden, connectivity between the right MTL seed and left posterior HC was significantly associated with better visual memory. RTLE with high disease burden was significantly correlated with decreased connectivity supportive of memory functions, between left MTL and bilateral anterior/posterior PHG, and between right MTL seed and bilateral inferior PG and right temporofrontal (middle TG and FG) cortex. Long epilepsy duration was significantly associated with weaker connectivity supportive of visual memory between right MTL seed and bilateral anterior PHG.

For verbal memory, RTLE patients with high disease burden showed less connectivity supportive of memory functions, between left MTL seed and left anterior PHG/FFG and right inferior/middle FG. RTLE with long epilepsy duration displayed weaker connectivity supportive of verbal memory between the left/right MTL seed and left/right OFC and between right MTL and right insula.

## 4 DISCUSSION

### 4.1 Task‐based functional memory network

On verbal and visual memory tasks, healthy controls displayed widespread functional connectivity between both MTL seeds and bilateral MTL regions, and bilateral temporoparietal cortices. Seeding from both MTLs, visual memory involved bilateral frontal connectivity (involving bilateral IFG and right OFC). For verbal memory, significant connectivity was apparent between the left MTL and left IFG, and at lower significance threshold, between the right MTL seed and left anterior HC and posterior FFG. Correlations of connectivity with memory performance showed that visual and verbal memory networks are supported by bilateral/contralateral MTL connectivity. We propose that the memory network involves a task‐based^37^ rather than material‐specific network, as suggested by previous activation‐based memory studies.^38^, ^39^, ^40^

Qualitatively, bilateral memory network representation was more prominent in RTLE than LTLE, as previously reported.^34^, ^41^ However, these studies examined functional connectivity at rest, reflecting state‐related networks, whereas the present findings extend this observation to memory‐related networks.

### 4.2 Widespread disruption within the memory network

As in previous studies^5^, ^24^, ^36^ clinical parameters such as seizure frequency, age at onset of epilepsy, epilepsy duration, and out‐of‐scanner memory scores are associated with differential patterns of memory network reorganization. We report functional connectivity changes controlling for these factors as regressors of no interest to explore network differences between controls and TLE as a disease syndrome.

Both TLE groups showed widespread connectivity changes, including decreased connectivity between MTL and frontal regions, and increased local connectivity to the left or bilateral anterior/posterior PHG than did controls. This pattern is consistent with a recent study in which resting‐state hyperconnectivity was shown close to the dysfunctional HC, and hypoconnectivity with remote cortical areas.^19^

#### 4.2.1 Reduced distant connectivity

For visual and verbal memory, there was no significant connectivity between either MTL seeds and frontal cortices in TLE, whereas healthy controls showed significant connectivity between both MTLs and bilateral/left IFG. Widespread extratemporal connectivity disruption may be related to structural^42^, ^43^ and functional abnormalities.^34^ Additionally, in TLE, intrinsic connectivity within MTL regions was significant mostly at lower threshold than that reported in controls, suggesting functional connectivity disruption in TLE. These findings support the network model of memory functions, whereby structural damage in one of the hubs (i.e., the MTL) compromises communication within the connected network. Previous studies suggest that verbal and visual memory deficits are seen after dominant and nondominant anterior temporal lobe resections, respectively.^44^, ^45^, ^46^, ^47^, ^48^ Nonetheless, there is accruing evidence of nonspecific memory decline after anterior temporal lobectomy.^49^, ^50^, ^51^, ^52^ Widespread network reorganization involving both MTLs, as reported herein, may provide an imaging biomarker for those who experience non‐material‐specific postoperative memory impairment. That is, greater extratemporal connectivity disruption may correlate with a greater risk of postoperative memory decline, irrespective of material type.^53^, ^54^

Patients with RTLE and healthy controls showed significant bilateral connectivity between MTL seeds and middle/superior TG for verbal and visual memory, whereas people with LTLE did not. For verbal memory, LTLE showed significant neocortical connectivity solely between the right MTL seed and the left TG. Qualitatively, the memory network in LTLE is more disrupted than in RTLE. This is consistent with previous evidence of more extensive functional^19^ and structural^55^, ^56^ network disruption associated with LTLE compared to RTLE. According to ontogenetic specialization, cognitive functions are represented bilaterally in the infant brain, and gradually lateralize with age.^57^, ^58^ The emergence of hemispheric specialization differs between hemispheres; the left hemisphere shows a slower and later network maturation. As such, early pathology to the left hemisphere may lead to a more diffuse memory network disruption than similar pathology in the more mature right hemisphere.

#### 4.2.2 Increased intrinsic MTL connectivity

Increased intrinsic functional connectivity within the anterior MTL was shown in both LTLE and RTLE compared to controls. In LTLE, increased anterior/posterior MTL functional connectivity was ipsilesional for visual memory, seeding from both MTLs, and bilateral for verbal memory, seeding from the right MTL. Functional reorganization analyses showed that LTLE subjects have reduced connectivity projecting from/to the left MTL that is supportive of memory functions, compared to controls. LTLE showed decreased connectivity between left MTL seed and right anterior PHG/left posterior FFG and PHG (for visual/verbal memory), and the parietotemporal cortex (bilateral middle/superior TG and right supramarginal gyrus). Compared to controls, LTLE displayed increased connectivity within the right MTL (to right amygdala) that is supportive of visual memory, and from right MTL to left superior temporal pole, which supports verbal memory.

These findings may indicate that in LTLE, increased left MTL connectivity reflects an attempted compensation for the diseased MTL and reduced distant functional connectivity, which converges with previous research. Despite structural damage of the HC,^59^ this structure appears functionally hyperconnected.^20^ Consistent with this, increased structural connectivity despite hippocampal atrophy has been shown in people with epilepsy^60^, ^61^, ^62^ and Alzheimer disease.^63^, ^64^ We expand these findings by suggesting that left‐sided MTL hyperconnectivity represents an inefficient reorganization, whereas increased connectivity within/from the right MTL is supportive of visual and verbal memory functions.^11^, ^12^

In RTLE, there was increased connectivity particularly within the contralateral anterior MTL for both verbal and visual memory. Functional reorganization analyses indicated that in RTLE compared to controls, connectivity supportive of memory functions was disrupted from the anterior MTL seeds to posterior left HC and right PHG/FFG. Previous research highlighted an anterior–posterior MTL gradient that is involved during encoding and retrieval stages.^65^ Disruption of efficient connectivity along the anterior‐to‐posterior MTL axis in RTLE may account for an attempted compensation within the nondiseased anterior MTL.

#### 4.2.3 Increased neocortical connectivity

Increased functional connectivity between MTL and contralesional temporal/frontotemporal cortices was shown in LTLE and RTLE compared to controls, at an exploratory threshold of *p* < .001, uncorrected. The lateralization of this functional reorganization was contralateral in LTLE with increased connectivity between right MTL and right superior temporal pole for verbal memory only. In LTLE, qualitative group comparisons showed a great disruption of the verbal memory network, whereby there was no significant neocortical connectivity seeding from the diseased MTL, and there was intrinsic MTL connectivity within left/right MTL regions solely at lower significance thresholds.

Functional reorganization analyses showed that LTLE had increased connectivity that is supportive of verbal memory between right MTL and left superior temporal cortex, compared to controls. Connectivity from the right MTL seed to the temporal cortex may reflect an attempted compensation for reduced wider functional connectivity within the diseased hemisphere.

Patients with RTLE showed increased connectivity compared to controls between MTLs and left neocortex for visual and verbal memory; between the right MTL and left inferior TG for visual memory, and both MTL seeds and left frontal cortex and insula for verbal memory. Sidhu et al. previously showed contralateral MTL activations during memory encoding in both LTLE and RTLE groups, which was associated with successful subsequent memory performance.^11^ We extend these findings by suggesting that memory functions are supported not only by contralateral activations, but by a network that includes connections between bilateral MTL regions, along with contralateral temporal and extratemporal regions. This may represent a functional compensation for the disrupted extratemporal functional connectivity, especially seeding from the diseased MTL.

#### 4.2.4 TLE due to HS is associated with more widespread memory connectivity disruption compared to TLE without HS

In general, TLE with HS showed increased intrinsic connectivity within anterior/posterior MTL regions, compared to those with TLE without HS. Increased intrinsic connectivity was noted primarily within the right anterior MTL in LTLE, and between both left and right posterior MTLs in RTLE, suggesting a differential functional compensation for the sclerotic MTL.

RTLE with HS compared to those without HS showed functional disruption with bilateral temporal cortices and left insula, areas previously associated with better memory performance.^11^

In TLE due to HS, structural MRI showed thalamic connectivity^66^ differences and functional imaging using arterial spin labeling^67^ showed differences in global network measures compared to those without HS suggesting a more disconnected functional brain network in people with TLE due to HS. No memory functional connectivity studies to date have described the memory network differences in TLE with and without HS. Some studies showed no impact of the type of pathology causing TLE to influence memory function,^68^ suggesting that the common focal epileptogenesis may be more associated with mnemonic functions in TLE. Nonlesional TLE, however, has been associated with better preoperative memory function and a greater risk of memory decline after epilepsy surgery.^69^

At an exploratory threshold level (*p* < .001, uncorrected), we report greater memory network disruption beyond the epileptogenic MTL in people with HS, which is in keeping with the greater connectivity disruption shown using other structural and functional imaging modalities in people with TLE due to HS.

### 4.3 Relation between functional connectivity and memory performance

We showed that increased connectivity within the anterior and posterior MTL structures were related to better memory in both groups, providing evidence of efficient functional reorganization of the memory network. Previously, Sidhu et al.^11^ showed that posterior hippocampal activations were associated with more successful memory in TLE. We propose that this region is a critical hub within the memory network, and that memory functions do not solely depend on its isolated activation, but rather are supported by local connectivity bilaterally.

This increased connectivity may strengthen the communication between bilateral posterior hippocampi, possibly to compensate for memory deficits, as suggested by Li et al.,^21^ who showed reorganization of intrahemispheric connectivity across the anterior and posterior hippocampal networks. Increased local functional connectivity within the MTL, despite structural damage, supports memory function and reflects cognitive efficiency of the memory network.

In both TLE groups, people with worse verbal memory performance showed higher connectivity between the pathological MTL and neocortical regions (at an exploratory threshold of *p* < .001, uncorrected). Particularly for verbal memory, increased connectivity to the right superior FG in LTLE, and to the left superior TG and right supramarginal gyrus in RTLE was associated with worse verbal memory performance, suggesting that this neocortical network reorganization is less efficient than intrinsic MTL connectivity.

Functional reorganization is not always effective, and may reflect inefficient attempts to compensate,^19^, ^41^ highlighting the importance of studying the implications of memory‐specific network reorganization for cognitive performance.

### 4.4 Clinical factors affecting reorganization of memory networks and their relationship with memory performance

Longer epilepsy duration was correlated with weaker connectivity between both MTL seeds and bilateral anterior/posterior MTL in RTLE, for visual and verbal memory. Higher seizure burden in RTLE, and longer epilepsy duration in LTLE, were associated with reduced connectivity to the left MTL. Further analyses showed that RTLE with greater disease burden have decreased connectivity supportive of visual memory between both MTLs and bilateral PHG, and for verbal memory, between left MTL and left anterior PHG. In LTLE, longer epilepsy duration was associated with reduced connectivity between left MTL seed and left anterior PHG for verbal memory. With regard to connectivity supportive of memory functions, LTLE with greater disease burden was correlated with decreased connectivity between both MTL seeds and bilateral MTL (bilateral HC and left FFG) for verbal memory, and from right MTL to left HC/PHG and to right amygdala for visual memory. These findings suggest that in TLE, a higher disease burden is associated with less efficient network reorganization, involving reduced connectivity to critical MTL regions, between both MTLs for visual memory and mainly within the left MTL for verbal memory.

Extratemporally, at an exploratory threshold of *p* < .001, uncorrected, for both visual and verbal memory, higher seizure burden and longer epilepsy duration were associated with disrupted connectivity between MTL seeds and OFC and IFG. Of note, measures of network organization in TLE have shown that greater integration and centrality of the left inferior frontal cortex preoperatively are predictive of better memory performance postoperatively.^70^, ^71^ Greater disease burden was correlated to decreased connectivity supportive of memory functions, between left/right MTL seeds and left/right OFC in RTLE, and in TLE between left/right MTL and right middle FG and left/right insula. This connectivity pattern is seen in healthy controls and comprises key regions for better memory performance in TLE.^11^, ^12^ Finally, TLE with greater disease burden showed weaker connectivity supportive of visual memory functions between right MTL and right middle TG, and connectivity disruption to the left middle TG was seen in RTLE with greater seizure frequency. Lower centrality of the left middle TG preoperatively is predictive of poorer memory performance postoperatively.^70^ Hence, neurobiologically, these findings support the view that earlier surgical intervention may be associated with a lower risk of memory decline postoperatively.

In TLE, earlier age at onset was correlated with increased connectivity within the posterior MTL, and reduced connectivity to extratemporal regions. Longer duration of epilepsy and higher seizure burden are associated with poorer memory performance in TLE.^72^ Here, we provide a biomarker for this association, represented by the disruption of functional connectivity between MTL and key extratemporal regions.

### 4.5 Limitations

Measures of seizure frequency were derived from patients' clinical histories and may not reflect true seizure burden. All patients were on antiepileptic drugs at the time of the study, and the effect of medications on the memory encoding network was not accounted for in these analyses. PPI analyses provide measures of functional connectivity, but do not allow inferences about the direction of the connection. PPI identifies task‐specific increases in the relationship between a seed region (i.e., the MTL in our study) and the rest of the brain. However, there is no implication that the seed region is the driver of the connection, and neocortical connectivity was reported at an exploratory threshold of *p* < .001, uncorrected. Finally, a visual check of the peak voxel with the 6‐mm radius ensured that the entire ROI was within the MTL for all four seed regions. However, there may still be cerebrospinal fluid and white matter confounds.

## 5 CONCLUSIONS

Functional connectivity measures derived from PPI are sensitive to seizure network effects. With this ROI‐to‐whole‐brain connectivity analysis, we demonstrated that the affected network extends beyond the epileptogenic temporal lobe, involving functional disruption in the contralateral temporal lobe as well as extratemporal regions. Higher seizure frequency and longer epilepsy duration were associated with connectivity disruption in the left/right OFC and insula, key regions for memory functions. Increased bilateral MTL connectivity represented efficient reorganization in people with epilepsy, and especially within the contralesional MTL in patients with LTLE.

Our findings highlight the richness of task‐based functional connectivity to investigate mnemonic function. Further investigations are required to understand how these aberrant and efficient functional networks relate to changes in structural connectivity between those same brain regions.

## AUTHOR CONTRIBUTIONS

Marine Fleury: Statistical analysis, data interpretation, and drafting and revision of manuscript. Sarah Buck: Statistical analysis, data interpretation, and drafting of manuscript. Lawrence P. Binding: Data analysis support and manuscript revision. Lorenzo Caciagli: Data interpretation, and manuscript revision and approval. Sjoerd B. Vos: Physics support, and manuscript revision and approval. Gavin P. Winston: Data collection, and manuscript revision and approval. Pamela J. Thompson: Data collection (clinical neuropsychological testing), and manuscript revision and approval. Matthias J. Koepp: Manuscript revision and approval. John S. Duncan: Interpretation, and manuscript revision and approval. Meneka K. Sidhu: Conception and design, data collection and interpretation, initial critical review, and manuscript revision and approval.

## FUNDING INFORMATION

This work was supported by the National Institute for Health Research University College London Hospitals Biomedical Research Centre (UCLH BRC; grant number 229811), The Wellcome Trust (grant number 083148), the Wellcome Trust Innovation Program (106882/Z/15/Z and 218380/Z/19/Z), and the Medical Research Council (G0802012, MR/M00841X/1). L.C. acknowledges support from a Brain Research UK PhD scholarship (award 14181). M.K.S. is supported by the UCLH BRC and Epilepsy Society.

## CONFLICT OF INTEREST

None of the authors has any conflict of interest to disclose. We confirm that we have read the Journal's position on issues involved in ethical publication and affirm that this report is consistent with those guidelines.

## Supporting information


Table S1
