# Supplementary material for: Episodic memory network connectivity in temporal lobe epilepsy
Source: Epilepsia. 2022 Aug 2;63(10):2597–622. doi: 10.1111/epi.17370 (PMC9804196; doi:10.1111/epi.17370)
Supplement: Supplementary file 1 — Table S1 [file EPI-63-2597-s001.docx]

*Table S1: Out-scanner Recognition Accuracy of In-scanner Verbal and Visual Memory Tasks*

|  | **Average** (*SD*) | |
| --- | --- | --- |
|  | Faces Remembered | Words Remembered |
| *Controls* | 28 (*12*) | 75 (*12*) |
| *LTLE* | 16 (*10*) | 53 (*21*) |
| *RTLE* | 14 (*9*) | 60 (*19*) |

LTLE=Left Temporal Lobe Epilepsy, RTLE = Right Temporal Lobe Epilepsy, SD = standard deviation. For each in-scanner memory task (words and faces encoding), out-scanner recognition accuracy is presented for each participant groups as mean (SD), with the highest accuracy score possible being 100. Group standard deviations are shown in brackets. Recognition accuracy was calculated as (true positives – false positives).
